# Are we overlooking the qualitative ‘look' of obesity?

**DOI:** 10.1038/nutd.2015.25

**Published:** 2015-07-20

**Authors:** A Perez, G D C Ball

**Affiliations:** 1 Department of Pediatrics, Faculty of Medicine and Dentistry, University of Alberta, Edmonton, Alberta, Canada

During the opening ceremonies of the 4th Canadian Obesity Summit held recently in Toronto, along with the traditional speeches and awards, a woman who formerly had obesity shared her personal story. Emotional, heart-felt, and humanizing, her experience of living with obesity as a child, a professional, a wife, and an artist provided a detailed and personal view of her ongoing personal struggles with her weight, which set the tone for the meeting over the next few days. Her story and others like it can provide rich insight into individuals' perspectives of obesity and weight management. In our view, these perspectives have been under-represented in the field of obesity research where numbers from quantitative research often take precedence over meanings derived from qualitative inquiry.

Qualitative research has proved important in many areas of clinical and health research, including understanding patients' and clinicians' decision making and enhancing quality of health services delivery related to utilization, feasibility and appropriateness of care.^1, 2^ Despite being on the rise, the publication of qualitative studies in medical journals is still low,^3^ especially in high-impact journals.^4^ This pattern is of concern given the role that high-impact journals have in disseminating new evidence to academic and clinical audiences^5^ as well as to the public through knowledge translation activities that follow publication, including both traditional (newspaper, television and radio) and social (Twitter, blogs) media outlets. Obesity research is not immune to this tendency. Recently, we completed an online search of original manuscripts published from January 2012 to December 2014 in five obesity journals (*Childhood Obesity*, *Clinical Obesity*, *International Journal of Obesity*, *Obesity*, and *Pediatric Obesity*). Of the total number of papers published (*n*=1961), qualitative reports comprised 1.1% (*n*=21). We also reviewed the authorship guidelines for all five journals and found no explicit statements regarding the exclusion of qualitative research or specific preferences for quantitative research, although some details (for example, testing hypotheses; including controls) were applicable to quantitative study designs only.

A search of bibliographic databases including PubMed and Scopus with obesity and qualitative research as key words yields hundreds of publications per year over the past several years, so a low total volume of qualitative studies related to obesity may not be a primary factor for the under representation of qualitative reports in obesity journals. That said, the poor quality and novelty of qualitative manuscripts submitted to obesity journals may be an issue; however, the extent to which this factor has a role is difficult to determine given that details regarding editors' and reviewers' familiarity and expertise in qualitative research are required along with the criteria used to gauge manuscript quality and appropriateness. As health research has been predominantly quantitative,^6^ the low proportion of qualitative studies published in obesity journals may not relate to poor quality, but to a lack of understanding, making it difficult for editors and reviewers to judge the value and quality of qualitative reports.

The tension between qualitative and quantitative research approaches and different underlying epistemologies have been documented.^7^ In our experience leading qualitative, obesity-related research with clinical and health services foci, we have gained some experience in addressing potential challenges with publication. For instance, the submission and resubmission processes provide opportunities for authors to include additional rationale for key methodological decisions, especially in relation to issues including sample size, hypothesis testing, reliability of coding, data saturation and generalizability of findings. Reviewers of our manuscripts have, more often than not, appreciated our explanations. This experience highlights the value of describing differences and addressing potential misperceptions between quantitative and qualitative research during the peer-review process. In addition, the inclusion of quality assessment and reporting checklists (for example,^8, 9^) that accompany our manuscript submissions has allowed us to provide a better understanding of the design, implementation, analysis and implications of our research. Using checklists to explain methodological and reporting details of qualitative studies may also benefit from a halo effect as it is consistent with many journal requirements for quantitative research.^10^ We have also been flexible in accommodating editors' and reviewers' recommendations to edit our manuscripts in circumstances when changes have not compromised the rigor of our qualitative research, but may not necessarily be consistent with the original intent of the research method (for example, including frequency data in thematic analyses).

We also believe that several steps can be taken to enhance the presence of qualitative research in obesity research. First, consistent with the approaches taken by other organizations (for example Canadian Obesity Network; Obesity Action Coalition), the inclusion and participation of individuals living with obesity at academic meetings can enable conversations about obesity in a manner that is less stigmatizing and biased, encouraging research (qualitative and quantitative) that is relevant to people living with obesity and providing a forum for their perspectives to inform research. Second, obesity journals can establish dedicated sections to highlight excellence in qualitative research, an approach that has been used to organize other research areas (for example,Clinical Trials and Investigations; Epidemiology/Genetics). Finally, the inclusion of explicit instructions within authorship guidelines for obesity journals can highlight the range of research considered for publication, which can include requiring applicable reporting checklists and be accompanied by the inclusion of scientists, clinicians, and administrators at all stages of the peer-review process who possess methodological expertise in both quantitative and qualitative research. The publication of qualitative research has been positively linked with journals' policies, authorship guidelines, and editorial content,^3^ so a proactive approach can be beneficial. Collectively, these steps can encourage more inclusive discussions about obesity as well as provide academic venues for publishing and disseminating research of greater epistemological breadth and relevance.
